# Surgical site infections following oral cavity cancer resection and reconstruction is a risk factor for plate exposure

**DOI:** 10.1186/s40463-017-0206-2

**Published:** 2017-04-08

**Authors:** Christopher M. Yao, Hedyeh Ziai, Gordon Tsang, Andrea Copeland, Dale Brown, Jonathan C. Irish, Ralph W. Gilbert, David P. Goldstein, Patrick J. Gullane, John R. de Almeida

**Affiliations:** Department of Otolaryngology-Head and Neck Surgery/Surgical Oncology, Princess Margaret Cancer Centre, University Health Network, 610 University Avenue, 3-955, Toronto, ON M5G 2 M9 Canada

**Keywords:** Surgical Site Infections, Plate-related Complications, Head and neck cancer, Plate exposure, Plate height, Mandibular reconstruction

## Abstract

**Background:**

Plate-related complications following head and neck cancer ablation and reconstruction remains a challenging problem often requiring further management and reconstructive surgeries. We aim to identify an association between surgical site infections (SSI) and plate exposure.

**Methods:**

A retrospective study between 1997 and 2014 was performed to study the association between postoperative SSI and plate exposures. Eligible patients included those with a history of oral squamous cell carcinoma who underwent surgical resection, neck dissection, and free tissue reconstruction. Demographic and treatment related information was collected. SSI were classified based on CDC definition and previously published literature. Univariable analysis on demographic factors, smoking history, diabetes, radiation, surgical and hardware related factors; while multivariable analysis on SSI, plate height, segmental mandibulectomy defects and radiation were conducted such as using cox proportional hazard models.

**Results:**

Three hundred sixty-five patients were identified and included in our study. The mean age of the study group was 59.2 (+/−13.8), with a predominance of male patients (61.9%). 10.7% of our patient cohort had diabetes, and another 63.8% had post-operative radiation therapy. Patients with SSI were more likely to have plate exposure (25 vs. 6.4%, *p* <0.001). Post-operative SSI, mandibulectomy defects, and plate profile/thickness were associated with plate exposure on univariable analysis (OR = 5.72, *p* < 0.001; OR = 2.56, *p* = 0.014; OR = 1.44, *p* = 0.003 respectively) and multivariable analysis (OR = 5.13, *p* < 0.001; OR = 1.36, *p* = 0.017; OR = 2.58, *p* = 0.02 respectively).

**Conclusion:**

Surgical site infections are associated with higher rates of plate exposure. Plate exposure may require multiple procedures to manage and occasionally free flap reconstruction.

## Background

Instrumentation with titanium plates is often required following ablative surgery for oral cancer. These plates are typically used for patients who require instrumentation for the surgical approach (e.g. mandibulotomy) or for reconstruction of mandibular defects. Plate-related complications may occur in up to 0–45% of cases, and may include plate exposure (4–46%), loose screws (0.8–5.8%), or plate fractures (0–3.3%) [1–16]. These complications may result in significant health care burden such as prolonged antibiotic therapy, revision surgery and impact patients’ quality of life.

Surgical site infections (SSIs) following head and neck cancer surgery may occur in as many as 10–45% of cases despite antibiotic prophylaxis [17–24]. SSIs have been defined by the Center for Disease Control and Prevention (CDC) as infection within the first 30 postoperative days with at least one of several factors, including purulent drainage, positive culture, and either a deliberate incision and drainage or presence of supporting signs and symptoms [25]. The development of SSIs can further lead to serious complications including wound breakdown, mucocutaneous fistulae, sepsis, and death. Delayed wound healing may also result in a poor cosmetic outcome, delayed oral intake and a delay in adjuvant therapies.

Several factors have been previously shown to be associated with the development of plate-related complications including plate related factors (plate material, plate profile, type and size of screws) [2, 4, 5], patient factors (smoking, diabetes, previous radiation, previous hyperbaric oxygen) [8, 9], and surgical defect [7, 10, 15]. We hypothesize that SSIs may result in colonization of the alloplastic plate and result in subsequent plate exposure. The present study aims to understand the relationship between post-operative surgical site infections and plate-related complications.

## Methods

Approval from the institutional review ethics board of the University Health Network was obtained. All patients 18 years or older who underwent an oral cavity resection and neck dissection for squamous cell carcinoma, requiring either a mandibulotomy or mandibulectomy with free flap reconstruction and osseous plating performed at the University Health Network in Toronto, Canada between 1997 and 2014 were identified. Eligible patients were identified using a pre-existing oral cavity database based off of the Cancer Registry from Princess Margaret Cancer Centre. Electronic medical records were reviewed to confirm candidacy. Patients who were treated with transoral approaches (i.e. no hardware used), or those requiring surgical management of osteoradionecrosis, and those with incomplete documentation of follow-up postoperative care were excluded.

All included patients received antimicrobial prophylaxis with cephalosporins (or clindamycin, if patient was documented with a penicillin allergy), and flagyl starting 30–60 min prior to incision and continuing for at least 24 h after surgery, although practices varied by practitioner. Surgical sites were sterilized prior to initial incision with either povidone-iodine or chlorhexidine.

Clinical information was ascertained from the electronic medical record, and paper charts for the early study period. Patient demographic information and comorbidities, treatment details, pathologic features, and oncologic outcomes were recorded. Postoperative wound infections were defined according to the Centers for Disease Control and Prevention (CDC) National Nosocomial Infections Surveillance (NNIS) system for superficial and deep incisional SSI, by criteria for post-operative wound infection following head and neck cancer surgery as described by Grandis et. al; and further included the development of an orocutaneous fistula in the presence of other infectious signs and symptoms (Table 1) [17, 25]. Distant infections such as pneumonia, or urinary tract infections were not captured in our study. Post-operative clinical notes were reviewed, and data pertaining to fevers, white count, differential, cultures, use of antibiotics, procedures including surgical debridement or incision and drainage at the bedside or in the operating room, presence of hematoma or hemorrhage were extracted. Furthermore, plate related characteristics including plate thickness, use of rescue screws, and use of locking screws were recorded. Surgical defects were categorized according to the bony and soft tissue defect. Bony defects were categorized as segmental or non-segmental mandibulectomy defects. Soft tissue defects were considered adverse if the defect involved the external skin, lip, buccal mucosa, mandibular alveolus, or retromolar trigone; sites where soft tissue resection places patients at a higher risk for plate related complications such as plate exposure. Other early post-operative wound related complications such as wound dehiscence, or flap compromise were also collected. Plate related complications (plate exposure, plate fracture) over the course of clinical follow-up were identified from clinical and operative notes. Loose screws were not captured in this study.

**Table 1:** Criteria for Surgical Site Infection

**CDC Guidelines**	**Grandis et al. 1992** [17]
**Superficial SSI:** Infection within 30 days of the operation Involving Skin and Subcutaneous tissue of the incision	Presence of fever, elevated leukocyte count, appearance of wound, institution of antimicrobial therapy
*At least one of:* a. Purulent drainage from the incision b. Organisms identified by aseptically obtained sample c. Incision is deliberately opened by a physician AND patient has at least one of the following: pain, localized swelling, erythema or heat d. Diagnosis of SSI by physician	
*The following are not included:* a. Stitch abscess alone b. The diagnosis and treatment of cellulitis (erythema, warmth, swelling) alone does not meet criteria	
**Deep SSI:** Infection within 30–90 days of the operation Involves the deeper soft tissues of the incision	
*At least one of:* a. Purulent drainage b. Deep incision with spontaneous dehiscence, or is deliberately opened by surgeon and organism is cultured and patient has at least one of the following signs and symptoms: fever, localized pain, and tenderness. c. Abscess, or radiological evidence of an infection.	

Patient demographic, treatment, and pathologic data were summarized using descriptive statistics. Univariable analysis determining the association between wound infection and plate-related complication was performed using cox proportional hazard ratios. Multivariable analyses using cox regression analysis was performed to account for the impact of other variables including plate height, segmental mandibulectomy defects, post-operative infection, and post-operative radiation.

## Results

A total of 365 patients meeting our study criteria were identified. The mean age of the study group was 59.2 (+/−13.8), with more males (61.9%) than females (38.1%) (Table 2). A hundred and two patients (27.9%) were actively smoking at the time of diagnosis, 111 (30.4%) had a history of smoking, and some never having smoked (36.7%). Only 10.7% of our patient cohort had diabetes, and another 63.8% had post-operative radiation therapy. Patients were reconstructed with either osseous-cutaneous free flaps (58.0%), or soft-tissue free flaps (39.2%), with one patient reconstructed using a pectoralis major (0.3%). Eighty-four patients (23.0%) developed surgical site infections within 30 days of their operation. The most common SSI formed were neck abscesses (11.5%), and orocutaneous fistulae (10%). Patient were followed for an average of 25.2 months.

**Table 2:** Demographics and patient characteristics of 365 patients

	Overall (365)	Infection (84)	No Infection (281)	*P*-Value
Age	59.2 (18.5 – 93.0)	59.5 (+/− 13.7)	59.1 (+/− 13.0)	0.853
Missing	0			
Sex				
M	226 (61.9%)	50 (59.5%)	176 (62.6%)	0.611
F	139 (38.1%)	34 (40.5%)	105 (37.4%)	
Missing	0			
Smoking				
non-smoker	134 (36.7%)	25 (29.8%)	109 (38.8%)	0.272
Ex-smoker	111 (30.4%)	32 (38.1%)	79 (28.1%)	
Active smoker	102 (27.9%)	22 (26.2%)	80 (28.5%)	
Missing	18 (4.9%)	5 (8.3%)	13 (4.6%)	
T2DM				
yes	39 (10.7%)	10 (11.9%)	29 (10.3%)	0.794
no	325 (89.0%)	74 (88.1%)	251 (89.3%)	
missing	1 (0.3%)	0	1 (0.4%)	
Plate Factors:				
Plate Size				
10 mm	10 (2.6%)	5 (5.5%)	5 (1.7%)	0.031
15 mm	279 (72.7%)	67 (73.6%)	212 (72.4%)	
20 mm	6 (1.6%)	2 (2.2%)	4 (1.4%)	
24 mm	16 (4.2%)	7(7.7%)	9 (3.1%)	
28 mm	14 (3.6%)	1(1.1%)	13 (4.4%)	
missing	59 (15.4%)	9 (9.9%)	50 (17.1%)	
Post-op Rads				
yes	233 (63.8%)	49 (58.3%)	184 (65.5%)	0.005
no	129 (35.3%)	32 (38.1%)	97 (34.5%)	
Missing	3 (0.8%)	3 (3.6%)		
Screws				
Locking	62 (17.0%)	9 (10.7%)	53 (18.9%)	0.106
Non-locking	247 (67.7%)	66 (78.6%)	181 (64.4%)	
Missing	56 (15.3%)	9 (10.7%)	47 (16.7%)	
Rescues	76 (20.8%)	18 (21.4%)	58 (20.6%)	0.618
Non-rescue	234 (64.1%)	57 (67.9%)	177 (63.0%)	
Missing	55 (15.1%)	9 (10.7%)	46 (16.4%)	
Surgical Defect:				
Soft Tissue:				
adverse^a^	179 (49.0%)	45 (53.6%)	135 (48.0%)	0.162
non-adverse	180 (49.3%)	36 (42.9%)	143 (50.9%)	
missing	6 (1.7%)	3 (3.5%)	3 (1.1%)	
Segmental Mandibulectomy Defect:				
Yes	212 (58.1%)	44 (52.4%)	168 (59.8%)	0.482
No	149 (40.8%)	39 (46.4%)	110 (39.1%)	
missing	4 (1.1%)	1 (1.2%)	3 (1.1%)	
Flaps				
Osseous +/− cutaneous	212 (58.0%)	40 (47.6%)	172 (61.2%)	0.426
Soft Tissue	143 (39.2%)	41 (48.8%)	102 (36.3%)	
Local Regional	1 (0.3%)	1 (1.2%)	0 (0.0%)	
Missing	9 (2.5%)	2 (2.4%)	7 (2.5%)	
Follow-up time (Median)	25.2 months	11.1 +/− 27.6 months	30.84 +/− 31.3 months	0.005
Plate Exposure				
yes	39 (10.7%)	21 (25.0%)	18 (6.4%)	<0.001
no	324 (88.8%)	63(75.0%)	261 (92.9%)	
missing	2 (0.5%)		2 (0.7%)	

^a^Adverse soft-tissue defects refer to surgical defects involving the retromolar trigone, buccal mucosa, mandibular alveolus, lip, and external skin

There were 39 (10.7%) patients who developed plate exposure post-operatively. There were no plate fractures in our population. Patients who developed post-operative SSI were more likely to develop subsequent plate exposure (25 vs. 6.4%, *p* <0.001). Univariable analysis performed on potential risk factors using Cox hazard ratio revealed post-operative infection (HR = 5.72, 95% CI = 3.04 – 10.80, *p* < 0.001), segmental mandibulectomy (HR = 2.56, 95% CI = 1.21 – 5.39, *p* = 0.014), and plate height (HR = 1.43, 95% CI = 1.13 – 1.82, *p* = 0.003) to be significantly associated with increased rates of plate exposures (Table 3). Patient characteristics such as age, sex, diabetes, post-operative radiation and smoking were not significantly associated. Other plate-related factors including use of rescue screw and locking screw; as well as adverse soft tissue defects were also not significantly associated.

**Table 3:** Univariate Analysis using Cox-Regression Analysis

Variable	Proportion of post-op exposure		Hazard ratio	95% CI	*P*-Value
	Exposure	No exposure			
Age					
<60 years	15 (4.1%)	145 (39.7%)	1.43	0.75 – 2.74	0.274
>60 years	24 (6.6%)	181 (49.6%)			
Sex					
male	27 (7.4%)	199 (54.5%)	0.674	0.341 – 1.331	0.255
female	12 (3.3%)	127 (34.8%)			
T2DM					
yes	4 (1.1%)	35 (9.6%)	1.051	0.373 – 2.957	0.925
no	35 (9.6%)	290 (79.5%)			
missing		1 (0.2%)			
Smoking					
active smoker	11 (3.0%)	91 (24.9%)	0.986	0.668 – 1.456	0.943
ex-smoker	12 (3.2%)	99 (27.1%)			
non-smoker	15 (4.1%)	119 (32.6%)			
missing	1 (0.2%)	17 (4.9%)			
Adj radiotherapy					
yes	28 (7.7%)	205 (56.2%)	1.461	0.727 – 2.940	0.287
no	11 (3.0%)	121 (33.1%)			
Use of rescue screw					
yes	14 (35.9%)	62 (19.0%)	1.132	0.849 – 1.510	0.398
no	24 (61.5%)	210 (64.4%)			
missing	1 (2.6%)	54 (16.6%)			
Use of locking screw					
yes	10 (25.6%)	52 (16.0%)	1.06	0.731 – 1.528	0.767
no	28 (71.8%)	219 (67.2%)			
missing	1 (2.6%)	55 (16.9%)			
Segmental Mandibulectomy					
yes	30 (76.9%)	182 (55.8%)	2.556	1.212 – 5.391	0.014
no	9 (23.1%)	140 (43.0%)			
missing		4 (1.2%)			
Adverse Soft Tissue					
yes	20 (51.3%)	159 (48.8%)	1.312	0.671 – 2.565	0.427
no	15 (38.5%)	165 (50.6%)			
missing	4 (10.2%)	2 (0.6%)			
Plate Height					
10 mm	3 (7.7%)	7 (2.1%)	1.436	1.131 – 1.824	0.003
15 mm	25 (64.1%)	236 (72.4%)			
20 mm	1 (2.6%)	5 (1.5%)			
24 mm	3 (7.7%)	12 (3.7%)			
28 mm	6 (15.3%)	8 (2.5%)			
missing	1 (2.6%)	58 (17.8%)			
Post-op Infection					
yes	21 (5.8%)	63 (17.2%)	5.72	3.04 – 10.80	<0.001
no	18 (4.9%)	263(72.1%)			

In multivariable analyses (Table 4), plate height, segmental mandibulectomy defects, SSI and post-operative radiation were included. SSI (HR = 5.13, 95% CI = 2.70 – 9.77, *p* <0.001), segmental mandibulectomy defects (HR = 2.58, 95% CI = 1.16 – 5.76, *p* = 0.020), and plate height (HR = 1.36, 95% CI = 1.06 –1.75, *p* = 0.017) were significantly associated with plate exposures in a Cox regression analysis. Post-operative radiation was not statistically associated with rates of plate exposure.

**Table 4:** Multivariate Analysis using Cox Regression Survival Analysis

Variables	Hazard radio	95% CI	*P*-Value
Post-op Infection	5.13	2.70 - 9.77	0.000
Segmental Mandibulectomy	2.58	1.16 – 5.76	0.020
Plate Height	1.36	1.06 – 1.75	0.017
Post-op Rads	1.02	0.47 – 2.13	0.996

**Table 5:** Management of 39 patients with plate exposure

Original Flap Utilized		
Fibular Flap	25 (64.1%)	
Radial Forearm Free Flap	7 (17.9%)	
Anterolateral Thigh Flap	3 (7.7%)	
Scapular Free Flap	4 (10.3%)	
Post-operative Issues:		
flap failures (24 h take-back)	3 (7.7%)	
infection	19 (48.7%)	
hematoma	1 (2.6%)	
Post-op Radiation:		
yes	26 (66.7%)	
no	13 (33.3%)	
Time to Plate Exposure:		
mean	15.1 months (0.4 – 120.8)	
median	9.24 months	
Exposure Location:		
intraoral	23 (59.0%)	
external	15 (38.5%)	
unknown	1 (2.5%)	
Mean Time to Plate Exposure by Location:		
Internal	13.6 +/− 10.4 months	*p* = 0.012*
External	42.3 +/− 18.0 months	
Concurrent Bony Concerns:		
non-union	7 (17.9%)	
bone exposure	5 (12.8%)	
Management:		
Conservative	11 (28.3%) (1 palliative, 1 complete closure, ongoing monitoring)	
OR Plate Removal/Debridement	9 (23.1%)	
OR Plate removal + Local Flap	6 (15.3%)	
OR Plate Removal + Free Flap	13 (33.3%)	
Outcomes:		
Multiple Revision	7 (17.9%)	
Chronic Drainage	1 (2.6%)	
Recurrence	2 (5.1%)	
Deceased	3 (7.7%)	

*calculated using student t-test

**Fig. 1:**
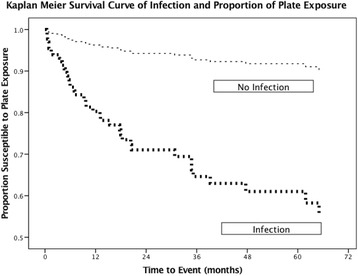
Kaplan Meier Survival Curve for Post-operative Infection and Proportion of Plate Exposure

The overall Kaplan-Meier curves for SSI and rates of plate exposure are displayed in Fig. 1. The 5-year probability of plate exposure free survival is 61.05 vs. 91.75%, (*p* <0.001) for patients with and without SSIs, respectively, as compared using the log-ranked test.

Majority of patients who developed plate exposure were initially reconstructed with bony osseous free flaps (74.4%) (Table 5). The overall mean time to plate exposure was 15.1 months. 59.0% of plate exposures occurred intra-orally, with 38.5% occurring externally, and 2.5% not documented. Plate exposures occurred intra-orally at a median time of 5.7 months compared with external plate exposures, which occurred at a median of 29.8 months. Twelve patients (30.7%) had concurrent bony concerns, with seven (17.9%) demonstrating non-union and five (12.8%) with concurrent bone exposure. No patients developed plate fractures in our study.

Management of these plate exposures included conservative approaches (11 patients, 28.3%), revision operations with plate removal and debridement of sequestra (9 patients, 23.1%), revision operations with plate removal and local flap (6 patients 15.3%), or revision operations with plate removal and free flap (13 patients, 33.3%) (Table 5). Of the patients managed with a free flap, 6 patients received a fibular free flap (46.2%), 4 patients received an anterolateral thigh free flap (30.8%), 2 received a radial forearm free flap (15.4%), and one received an unknown free flap (7.6%). Seven of these patients (17.9%) were re-plated after removal of the exposed plate. During the follow-up of these patients, another 7 patients (17.9%) required multiple procedures.

## Discussion

In the present study we showed a strong association between SSIs and plate-related complications. As no patient in our population had plate fractures, we focused on plate exposures. Plate profile as well as segmental mandibular defects reconstructed with osseous free flaps are also associated with plate exposures. The rates of post-operative SSI and plate exposures in the present study are corroborated by previous studies (26.8% compared with 22–46% [19, 24, 26, 27] and 12.3% compared with 4–46% [1–16]). To date, however, our study is the first that demonstrates an association between SSI and plate exposures.

There are several factors that have previously been established that are associated with plate complications. In the present study, we chose a homogenous population of patients with oral cavity squamous cell carcinoma. This patient population is associated with risk factors such as smoking that in and of themselves may predispose patients to impaired healing and subsequent plate complications [28]. Other non-surgical factors such as diabetes has been shown to significantly predict plate complications [9]. In our population, commonly held non-surgical risk factors for plate-related complications including smoking, diabetes, pre-operative or post-operative radiation, and chemotherapy, were not significantly associated with plate-exposures. Despite not being found to be independently significant for plate exposure, the significance of these risk factors cannot be overlooked given the well-established biological processes whereby these factors can impair wound healing [29–31].

Herein we describe a strong association between SSIs and plate exposures. Infections of the head and neck following ablative surgery may lead to bacterial colonization of plates, resulting in biofilm formation, wound contamination and subsequent plate exposure requiring hardware removal to eliminate the nidus of infection [32]. Durand et al. recently reviewed their experience of SSIs following head and neck free reconstructive surgeries reporting 25% of their swabs growing normal oral flora, 44% gram-negative bacilli, 20% methicillin-resistant *Staphylococcus aureus* and 16% methicillin-sensitive *Staphylococcus aureus* [33]. The authors found that in 67% of cultures, at least one pathogen was found to be resistant to prophylactic antibiotics. These infections that are often difficult to treat corroborate our finding that surgical site infections may lead to plate exposure as they are often recalcitrant to antimicrobial therapy.

Other studies focusing on the pathophysiology of plate exposures have previously suggested both plate material and plate profile to be potential predictors [1, 2, 4]. Although multiple studies have found no significant difference between stainless steel and titanium plates in complication rates, when lower profile plates were used, plate exposure rates were found to decrease from 20 to 4% [34, 35]. These studies corroborate our finding that higher profile plates were associated with increased plate exposure in both univariable and multivariable analysis.

Surgical defect size is another potential confounding factor that may be related to plate related complications. We showed that patients with segmental mandibulectomy defects are more likely to develop plate exposures. Although there are several existing classifications schemes for the reconstruction of mandibular defects that further categorize mandibulectomy defects, we chose to dichotomize this variable as the primary outcome was the association of infections with plate exposures [36–39].

Adequate reconstruction after ablative surgery with sufficient soft tissue restoration is critical in avoiding plate exposures. For patients with mandibulectomy defects, reconstruction with vascularized bone is imperative for anterior segmental defects to avoid an “Andy Gump” deformity while for patients with lateral defects some groups propose a soft tissue reconstruction with or without a plate as an alternative to vascularized bony reconstruction depending on overall disease prognosis, age, dentition, and comorbid status [15, 16, 40, 41]. Furthermore, with larger soft tissue defects, osseocutaneous flaps may not have adequate associated soft tissue components, and two free tissue transfers may be required to optimize the reconstruction, adding to both surgical time and complexity [41]. Whichever reconstruction method is chosen, if insufficient bone and soft tissue were used to reconstruct the defect, wound contracture and steady pressure of the plate against the skin may lead to eventual plate exposure [14]. In one study, over-reconstructing medial soft tissue aspects and obliterating dead space resulted in a reduction of plate exposures from 38 to 8% even in patients reconstructed with lateral defects with a plate and soft tissue [41]. The site of mandibulectomy defect was at one point considered an important factor in eventual plate exposure, with mandibulectomy defects involving the central mandible found to have higher rates of plate exposure [7]. With improved microvascular reconstructive techniques, however, the site of the mandibulectomy defect was not found to be a significant predictor of plate exposure [5, 8, 9]. Overall, studies have found lower rates of plate exposure in patients with mandibulotomies (0–15%) [42–45]. In the present study, we showed decreased plate exposure with mandibulotomies compared to those with mandibulectomy defects. This is likely due to the length of the plate in addition to the associated soft tissue defects.

Plate exposures continue to be the most common plate-related complication in mandibular reconstructive surgery [1–16]. Although in some instances managed conservatively, many plate exposures affect patient quality-of-life and plate removal with secondary reconstruction is occasionally necessary [3]. In our study, several patients required plate removal with secondary reconstruction. In addition, some patients develop recurrent plate exposures, suggesting that there may be systemic factors leading to poor wound healing.

Plate exposures can be classified as intra-oral or extra-oral. Nicholsen et al. noted a pattern where extra-oral plate exposure occurred at a mean of ten months post-operatively, while intra-oral plate exposure occurred at a mean of six weeks – three months [7]. This pattern was also seen in our population, with intraoral exposures occurring earlier than external exposures. Given the difference in timing, it is conceivable that the pathophysiology may differ between these two entities. Although there is little evidence to support this, we hypothesize that intraoral exposures are secondary to wound breakdown and salivary contamination whereas external exposure is likely related to longstanding pressure necrosis of the surrounding soft tissues although wound infection is still a contributing factor as we have seen in the present study.

Our study had several limitations. It is limited by a retrospective design albeit the findings of the association between SSI and plate exposure are strongly significant. Furthermore, some definitions used were subjective such as the definition of an adverse soft tissue defect. Furthermore, given the retrospective design, we were unable to study the volume of tissue extirpated and the volume of tissue reconstructive, both of which have implications on the development of plate exposures. Lastly the scope of our study did not capture several important outcome measures such as the impact of plate exposure on mastication, swallowing, speech, and quality of life. Future studies may address some of these issues.

## Conclusions

Mandibular reconstruction remains a challenging task for the head and neck reconstructive surgeon. Numerous factors including the defect size, location of the defect, and presence of wound healing compromising conditions must be judiciously reviewed and considered to prevent plate-related complications. SSIs may portend a greater risk towards the development of plate exposure, as does plate height and adverse bony defects. Plate exposure may require multiple procedures to manage and occasionally free flap reconstruction.

## Abbreviations

CDC: Centers for disease control and prevention
NNIS: National nosocomial infections surveillance
SSI: Surgical site infection

## References

[1] Futran ND, Urken ML, Buchbinder D, Moscoso JF, Biller HF (1995). Rigid fixation of vascularized grafts in mandibular reconstruction. Arch Otolaryngol Head Neck Surg.

[2] Klotch DW, Gal TJ, Gal RL (1999). Assessment of plate use for mandibular reconstruction: has changing technology made a difference?. Otolaryngol Head Neck Surg.

[3] Wei FC, Celik N, Yang WG, Chen IH (2003). Complications after reconstruction by plate and soft-tissue free flap in composite mandibular defects and secondary salvage reconstruction with osteocutaneous flap. Plast Reconstr Surg.

[4] Farwell DG, Kezirian EJ, Heydt JL, Yueh B, Futran ND (2006). Efficacy of small reconstruction plates in vascularized bone graft mandibular reconstruction. Head Neck.

[5] Knott PD, Suh JD, Nabili V, Sercarz JA, Head C, Abemayor E, Blackwell KE (2007). Evaluation of hardware-related complications in vascularized bone grafts with locking mandibular reconstruction plate fixation. Arch Otolaryngol Head Neck Surg.

[6] Zavattero E, Fasolis M, Garzino-Demo P, Berrone S, Ramieri GA (2014). Evaluation of plate-related complications and efficacy of fibula free flap mandibular reconstruction. J Craniofac Surg.

[7] Nicholson RE, Schuller DE, Forrest A (1997). Factors involved in long- and short-term mandibular plate exposure. Arch Otolaryngol Head Neck Surg.

[8] Maurer P, Eckert AW, Kriwalsky MS, Schubert J (2010). Scope and limitations of methods of mandibular reconstruction: a long-term follow-up. Br J Oral Maxillofac Surg.

[9] van der Rijt EE, Noorlag R, Koole R, Abbink JH, Rosenberg AJ (2015). Predictive factors for premature loss of Martin 2.7 mandibular reconstruction plates. Br J Oral Maxillofac Surg.

[10] Deleyiannis FWB, Rogers C, Lee E, Russavage J (2006). Reconstruction of the lateral mandibulectomy defect: management based on prognosis and location and volume of soft tissue resection. Laryngoscope.

[11] Boyd JB (1994). Use of reconstruction plates in conjunction with soft-tissue free flaps for oromandibular reconstruction. Clinic in Plast Surg.

[12] Urken ML, Buchbinder D, Costantino PD (1998). Oromandibular reconstruction using microvascular composite flaps: report of 210 cases. Arch Otolaryngol Head Neck Surg.

[13] Head C, Alam D, Sercarz JA (2003). Microvascular flap reconstruction of the mandible: a comparison of bone grafts and bridging plates for restoration of mandibular continuity. Otolaryngol Head Neck Surg.

[14] Onoda S, Kimata Y, Yamada K, Sugiyama N, Onoda T, Eguchi M, Mizukawa N (2012). Prevention points for plate exposure in the mandibular reconstruction. J Craniomaxillofac Surg.

[15] Arden RL, Rachel JD, Marks SC (1999). Volume-length impact of lateral jaw resections on complication rates. Arch Otolaryngol Head Neck Surg.

[16] Mariani PB, Kowalski LP, Magrin J (2006). Reconstruction of large defects postmandibulectomy for oral cancer using plates and myocutaneous flaps: a long-term follow-up. Int J Oral Maxillofac Surg.

[17] Grandis JR, Snyderman CH, Johnson JT, Yu VL, D’Amico F (1992). Postoperative wound infection – a poor prognostic sign for patients with head and neck cancer. Cancer.

[18] Girod DA, McCulloch TM, Tsue TT, Weymuller JREA (1995). Risk factors for complications in clean-contaminated head and neck surgical procedures. Head Neck.

[19] de Melo GM, Ribeiro KC, Kowalski LP, Deheinzelin D (2001). Risk factors for postoperative complications in oral cancer and their prognostic implications. Arch Otolaryngol Head Neck Surg.

[20] Penel N, Fournier C, Lefebvre D, Lefebve JL (2005). Multivariate analysis of risk factors for wound infection in head and neck squamous cell carcinoma surgery with opening of mucosa. Study of 260 surgical procedures. Oral Oncol.

[21] Simo R, French G (2006). The use of prophylactic antibiotics in head and neck oncologic surgery. Curr Opin Otolaryngol Head Neck Surg.

[22] Lotfi CJ, Cavalcanti Rde C, Silva AM C e, Latorre Mdo R (2008). Risk factors for surgical-site infections in head and neck cancer surgery. Otolaryngol Head Neck Surg.

[23] Ogihara H, Akeuchi K, Majima Y (2009). Risk factors of postoperative infection in head and neck surgery. Auris Nasus Larynx.

[24] Lee DH, Kim SY, Nam SY, Choi SH, Choi JW, Roh JL (2011). Risk factors of surgical site infection in patients undergoing major oncological surgery for head and neck cancer. Oral Oncol.

[25] Horan TC, Gaynes RP, Martone WJ, Jarvis WR, Emori TG (1992). CDC definitions of nosocomial surgical site infections, 1992: a modification of CDC definitions of surgical wound infections. Am J Infect Control.

[26] Mitchell RM, Mendez E, Schmitt NC, Bhrany AD, Futran ND (2015). Antibiotic prophylaxis in patients undergoing head and neck free flap reconstruction. JAMA Otolaryngol Head Neck Surg.

[27] Yang CH, Chew KY, Solomkin JS (2013). Surgical site infections among high-risk patients in clean-contaminated head and neck reconstructive surgery. Ann Plast Surg.

[28] Shuman AG, Entezami P, Chernin AS, Wallace NE, Taylor JMG, Hogikyan ND (2010). Demographics and efficacy of head and neck cancer screening. Otolaryngol Head Neck Surg.

[29] Brem H, Tomic-Canic M (2007). Cellular and molecular basis of wound healing in diabetes. J Clin Investig.

[30] Sorensen LT (2012). Wound healing and infection in surgery: the pathophysiological impact of smoking, smoking cessation, and nicotine replacement therapy: a systematic review. Ann Surg.

[31] Haubner F, Ohmann E, Pohl F, Strutz J, Gassner HG (2012). Wound healing after radiation therapy: review of the literature. Radiat Oncol.

[32] Brady RA, Leid JG, Calhoun JH, William Costerton J, Shirtliff ME (2008). Osteomyelitis and the role of biofilms in chronic infection. Pathog Dis.

[33] Durand ML, Yarlagadda BB, Rich DL, Lin DT, Emerick KS, Rocco JW, Deschler DG (2015). The time course and microbiology of surgical site infections after head neck free flap surgery. Laryngoscope.

[34] Blackwell KE, Buchbinder D, Urken ML (1996). Lateral mandibular reconstruction using soft-tissue free flaps and plates. Arch Otolaryngol Head Neck Surg.

[35] Blackwell KE, Lacombe V (1999). The bridging lateral mandibular reconstruction plate revisited. Arch Otolaryngol Head Neck Surg.

[36] Jewer DD, Boyd JB, Manktelow RT, Zuker RM, Rosen IB, Gullane PJ (1989). Orofacial and mandibular reconstruction with the iliac crest free flap: a review of 60 cases and a new method of classification. Plast Reconstr Surg.

[37] Urken ML, Weinberg H, Vickery C, Buchbinder D, Lawson W, Biller HF (1991). Oromandibular reconstruction using microvascular composite free flaps: report of 71 cases and a new classification scheme for bony, soft-tissue, and neurologic defects. Arch Otolaryngol Head Neck Surg.

[38] Iizuka T, Häfl iger J, Seto I, Rahal A, Mericske-Stern R, Smolka K (2005). Oral rehabilitation after mandibular reconstruction using an osteocutaneous fibula free flap with endosseous implants. Factors affecting the functional outcome in patients with oral cancer. Clin Oral Implants Res.

[39] Brown JS, Barry C, Ho M, Shaw R (2016). A new classification for mandibular defects after oncological resection. Lancet Oncol.

[40] Chim H, Salgado CJ, Mardini S, Chen HC (2010). Reconstruction of mandibular defects. Semin Plast Surg.

[41] Chepeha DB, Teknos TN, Fung K (2008). Lateral oromandibular defect: when is it appropriate to use a bridging reconstruction plate combined with a soft tissue revascularized flap?. Head Neck.

[42] Danan D, Mukherjee S, Jameson MJ, Shonkda DC (2014). Open reduction internal fixation for midline mandibulotomy: lag screws vs plates. JAMA Otolaryngol Head Neck Surg.

[43] Dubner S, Spiro RH (1991). Median mandibulotomy: a critical assessment. Head Neck.

[44] Amin MR, Deschler DG, Hayden RE (1999). Straight midline mandibulotomy revisited. Laryngoscope.

[45] Shinghal T, Bissada E, Chan HB, Wood RE, Atenafu EG, Brown DH, Gilbert RW, Gullane PJ, Irish JC, Waldron J, Goldstein DP (2013). Medial Mandibulotomies: Is there sufficient space in the midline to allow a mandibulotomy without compromising the dentition?. J Otolaryngol Head Neck Surg.

